# Lens-Free On-Chip Quantitative Phase Microscopy for Large Phase Objects Based on a Biplane Phase Retrieval Method

**DOI:** 10.3390/s25010003

**Published:** 2024-12-24

**Authors:** Yufan Chen, Xuejuan Wu, Yang Chen, Wenhui Lin, Haojie Gu, Yuzhen Zhang, Chao Zuo

**Affiliations:** 1 Smart Computational Imaging Laboratory (SCILab), School of Electronic and Optical Engineering, Nanjing University of Science and Technology, Nanjing 210094, Chinachenyang0108@njust.edu.cn (Y.C.);; 2 Smart Computational Imaging Research Institute (SCIRI), Nanjing University of Science and Technology, Nanjing 210019, China; 3 Jiangsu Key Laboratory of Spectral Imaging & Intelligent Sense, Nanjing 210094, China

**Keywords:** lens-free on-chip microscopy, quantitative phase imaging, biplane phase retrieval, high-throughput imaging, iterative phase retrieval

## Abstract

Lens-free on-chip microscopy (LFOCM) is a powerful computational imaging technology that combines high-throughput capabilities with cost efficiency. However, in LFOCM, the phase recovered by iterative phase retrieval techniques is generally wrapped into the range of −π to π, necessitating phase unwrapping to recover absolute phase distributions. Moreover, this unwrapping process is prone to errors, particularly in areas with large phase gradients or low spatial sampling, due to the absence of reliable initial guesses. To address these challenges, we propose a novel biplane phase retrieval (BPR) method that integrates phase unwrapping results obtained at different propagation distances to achieve accurate absolute phase reconstruction. The effectiveness of BPR is validated through live-cell imaging of HeLa cells, demonstrating improved quantitative phase imaging (QPI) accuracy when compared to conventional off-axis digital holographic microscopy. Furthermore, time-lapse imaging of COS-7 cells in vitro highlights the method’s robustness and capability for long-term quantitative analysis of large cell populations.

## 1. Introduction

Optical microscopes are indispensable tools in biomedical research and life sciences. However, traditional systems are limited by the Lagrange invariant, which imposes a trade-off between imaging resolution and field of view (FOV) [1,2], thereby restricting the spatial bandwidth product. Biological cells are often colorless and transparent, resulting in low contrast under brightfield illumination. To enhance contrast, staining or labeling techniques are commonly applied [3,4], which can potentially damage cells or interfere with live-cell observations. Quantitative phase imaging (QPI) [5,6,7] offers a powerful solution by enabling non-invasive visualization of internal structures and refractive index distributions in transparent and semi-transparent samples, making it ideal for live-cell imaging applications [8,9,10].

The recent development of computational microscopy technology [11,12,13,14,15] provides new opportunities for high-resolution QPI over a large FOV, synthetic-aperture interference microscopy [16,17,18], Fourier ptychographic microscopy (FPM) [19,20,21], transport of intensity equation (TIE) [22,23], differential phase contrast (DPC) [24,25,26], and lens-free on-chip microscopy (LFOCM) [27,28,29]. Among them, LFOCM has attracted the attention of researchers thanks to its simple optical design and low-cost equipment requirements. Compared to traditional microscopes, the main advantage of LFOCM is that its imaging FOV can reach the size of the photosensitive surface of the image sensor, making it a promising high-throughput QPI technology suitable for high-throughput imaging of subcellular structures in biological samples, including cell biology, digital pathology, and drug screening.

The LFOCM system consists of only an illumination source, a sample, and a sensor. It is based on the principle of in-line holography, where the incident coherent beam passes through the sample, the scattered beam interferes with the unscattered beam, and the hologram formed by the coherent superposition of the two beams is recorded by the sensor. The quantitative phase information of the sample is obtained by digital refocusing and phase reconstruction of the acquired diffraction pattern. The resolution of LFOCM systems is mainly limited by the sensor pixel size (typically larger than 1 µm) due to the under-sampling of the acquired holograms at unit fringe magnification. To overcome this limitation, a number of pixel super-resolution methods have been proposed, including sub-pixel displacement of the light source, sample, or sensor [30,31], active parallel plate scanning [32], axial scanning of the sample-to-sensor distance [33,34], and wavelength scanning [35,36,37], to break through the physical limitation of the sampling, corresponding to the sensor pixel size, and to obtain an extremely large spatial bandwidth product.

However, when imaging large phase objects with conventional LFOCM, the 2D transmittance function’s inaccurate modeling of thick 3D samples, combined with a low spatial sampling rate, leads to reconstruction errors. These errors ultimately manifest as black spots in the image. In existing works, the coded ptychographic imaging method [38], using coded image sensors and laser sources, has also been applied to the reconstruction of large phase objects. However, this method primarily focuses on large phase objects with slow-varying phase profiles and involves relatively complex setups. In contrast, achieving precise reconstruction of large phase samples using a miniaturized, lens-free microscope with an LED light source would offer significant advantages in terms of both simplicity and cost effectiveness. In this paper, we propose a biplane phase retrieval (BPR) algorithm for phase reconstruction of large phase objects. By using the BPR method of modeling the large phase objects with the two-slice approximation, we obtain the initial phase at different propagation distances [39] and combine it with subsequent iterative reconstruction methods to accurately reconstruct the phase of large phase objects. Considering wavelength-scanning-based LFOCM does not require complicated and time-consuming mechanical operations, we applied our method to it to verify the effectiveness of the BPR method. By comparing with the results from digital holography, we verified the accuracy of the QPI results, especially for samples with large phase values. Long-term observation experiments on COS-7 cells validated the robust quantitative phase reconstruction of various cell morphologies during growth and development, which demonstrates great potential for cell morphology and cell mass measurements.

## 2. Materials and Methods

### 2.1. System Setup

**Figure 1:**
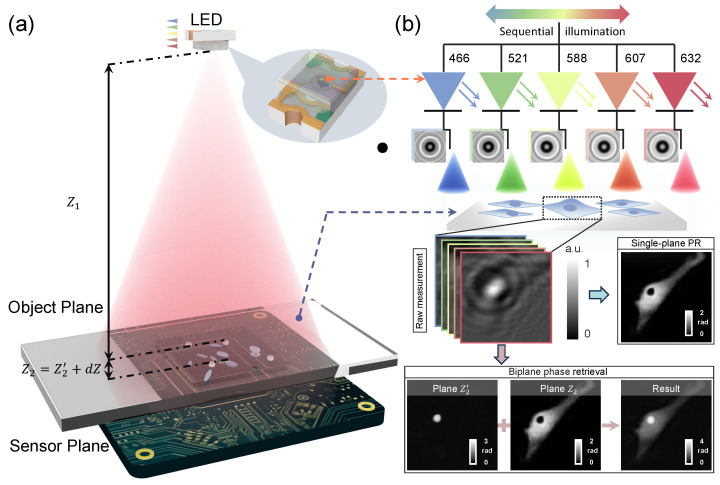
The BPR of lens-free on-chip quantitative phase microscopy. (**a**) Schematic of the LFOCM system, including the CMOS sensor, color LED matrix, and sample. (**b**) Schematic comparison of BPR and traditional single-plane phase retrieval (PR) method under multi-wavelength illumination.

The experimental setup employs a multi-wavelength scanning-based LFOCM system, illustrated in Figure 1a. The setup comprises two main components: a CMOS sensor (5664 × 4256 resolution; pixel size: 0.9 µm) and a color LED matrix. The LED matrix contains five quasi-monochromatic LEDs emitting wavelengths of 466 nm, 521 nm, 588 nm, 607 nm, and 632 nm, with bandwidths ranging from 20 to 50 nm. The refractive index of our samples in the 466–632 nm range is almost constant (Δn<0.35%) [40], so we disregard the effect of dispersion between various wavelengths of LED illumination. A self-developed circuit is used as a programmable power switch for the camera to prevent overheating of the sensor surface (which might damage the cells inside the dish). It also generates control signal sequences to synchronize the camera with the LED. The partially coherent beam travels $Z_{1}(∼$100 mm) to interact with the sample, generating in-line holograms. According to the incremental sequence $\left\{\lambda_{m},m=1,2,3,\ldots ,M\right\}$ (in this paper the number of wavelengths *M* = 5), the hologram $I^{m}$ at wavelength $\lambda_{m}$ is recorded by a CMOS sensor placed close to the sample ($Z_{2}∼$500 µm).

### 2.2. Sample Preparation

To assess the accuracy of phase reconstruction for large phase objects using the BPR method, three types of experimental samples were prepared for distinct validation experiments. These include mouse vascular endothelial cells (C166 cells) for slide-based imaging, human cervical cancer cells (HeLa cells) for QPI validation, and African green monkey kidney fibroblast cells (COS-7 cells) for live-cell culture studies. C166 cells are immersed and fixed in buffered glycerol to maintain their structural integrity and morphology. After fixation, these cells are meticulously processed into cell slides for use in an experiment titled “BPR-LFOCM on Cell Slides”. Similarly, HeLa cells and COS-7 cells are cultured in 20 mm glass-bottom dishes supplemented with 10% fetal bovine serum without any staining and labeling treatments. The HeLa cells were employed in “BPR-LFOCM on QPI Validation”, an experiment that verified the accuracy of BPR in QPI. Meanwhile, COS-7 cells were utilized in “BPR-LFOCM on Live Cell Culture”, aimed at imaging and monitoring long-term live cells.

### 2.3. Imaging Principle

**Figure 2:**
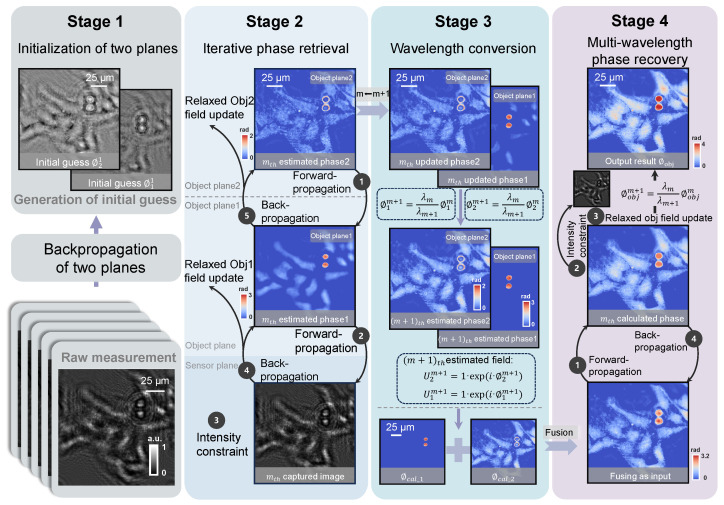
Flowchart of the BPR algorithm, illustrating four stages: initialization of two planes, iterative phase retrieval, wavelength conversion and fusion, and multi-wavelength phase recovery.

The reconstruction process is summarized in Figure 2, comprising four main stages. Stages 1 through 3 involve the generation and fusion of phase data from two planes, which serve as the initial input for Stage 4. The final stage performs multi-wavelength phase recovery to achieve accurate phase reconstruction.

Stage 1: Initialization of two planes. The BPR method models the large phase sample as dual layers with object plane 1 and object plane 2 at defocusing distances of $Z_{2}^{\prime }$ and $Z_{2}$, respectively. The captured hologram $I^{1}$ is back-propagated to the two object planes with the angular spectrum method to generate initial phase guesses $\phi_{1}^{1}$ and $\phi_{2}^{1}$ of the two object fields.

Stage 2: Iterative phase recovery. This is the key step in the reconstruction algorithm, which can be further divided into three sub-steps.

(1) Forward propagation. The uniform light intensity constraint is applied to the complex amplitude of the object plane 2 at the *m*th wavelength:

(1) $$U_{2}^{m}=\exp\left(j\phi_{2}^{m}\right)$$

The wave $U_{2}^{m}$ is then forward-propagated to object plane 1 to obtain the wavefront $U_{1a}^{m}$, which is next combined with the phase of $\phi_{1}^{m}$ to obtain the exit wave $U_{1b}^{m}$:

(2) $$U_{1b}^{m}=U_{1a}^{m}\exp\left(j\phi_{1}^{m}\right)$$

The exit wave $U_{1b}^{m}$ at object plane 1 is forward-propagated to the image plane to obtain the image field $U_{img}^{m}$.

(2) Relaxed image field update. The image field is updated in a recursive manner:

(3) $$U_{update}^{m}=\left(1-\alpha \right)U_{img}^{m}+\alpha \sqrt{I^{m}}\exp\left[j\arg\left(U_{img}^{m}\right)\right]$$

(3) Back-propagation. $U_{update}^{m}$ is back-propagated to the object plane 1 to obtain the updated exit wave $U_{update\_1b}^{m}$. The object field $U_{update\_1}^{m}$ and wavefront $U_{update\_1a}^{m}$ at object plane 1 are updated as follows:

(4) $$U_{update\_1}^{m}=\left(1-\beta \right)\exp\left(j\phi_{1}^{m}\right)+\beta U_{update\_1b}^{m}/U_{1a}^{m}$$

(5) $$U_{update\_1a}^{m}=U_{update\_1b}^{m}/U_{update\_1}^{m}$$

The updated wavefront $U_{update\_1a}^{m}$ at object plane 1 is back-propagated to the object plane 2 to obtain the updated exit wave $U_{update\_2b}^{m}$. The object field at object plane 2 is updated as follows:

(6) $$U_{update\_2}^{m}=\left(1-\gamma \right)\exp\left(j\phi_{2}^{m}\right)+\gamma U_{update\_2b}^{m}.$$

where arg(·) is the function to obtain the phase angle. α, β, and γ (typically set to less than one) are the constant parameters controlling the amount of feedback from the previous estimate. Within this research, we set the constant parameters α, β, and γ to 0.1.

Stage 3: Wavelength conversion and fusion. The phase component should be changed proportionally while changing the wavelength. Moreover, the wavelength conversion should be implemented on the unwrapped phase map, and additional two-dimensional phase unwrapping should be performed [41].

(7) $$\phi_{1}^{m+1}=\left(\lambda_{m}/\lambda_{m+1}\right)\phi_{1}^{m}$$

(8) $$\phi_{2}^{m+1}=\left(\lambda_{m}/\lambda_{m+1}\right)\phi_{2}^{m}$$

After wavelength conversion, the updated complex amplitude for the next sub-iteration is denoted as

(9) $$U_{1}^{m+1}=\exp\left(j\phi_{1}^{m+1}\right)$$

(10) $$U_{2}^{m+1}=\exp\left(j\phi_{2}^{m+1}\right)$$

Stages 2 and 3 are then repeated *M* times until all wavelengths have been used (all raw holograms have been processed once). The phases of the two planes obtained after iterative convergence are fused.

Stage 4: Multi-wavelength phase recovery [37]. The fused complex amplitude is used as the initial input for the multi-wavelength phase recovery algorithm to obtain an accurate phase reconstruction.

The stage 4 reconstruction process typically requires 10–20 iterations to converge (depending on the sample complexity and data quality), and the resultant $\phi_{obj}$ provides the phase distribution of the measured object.

## 3. Experiments and Results

### 3.1. BPR-LFOCM on Cell Slides

**Figure 3:**
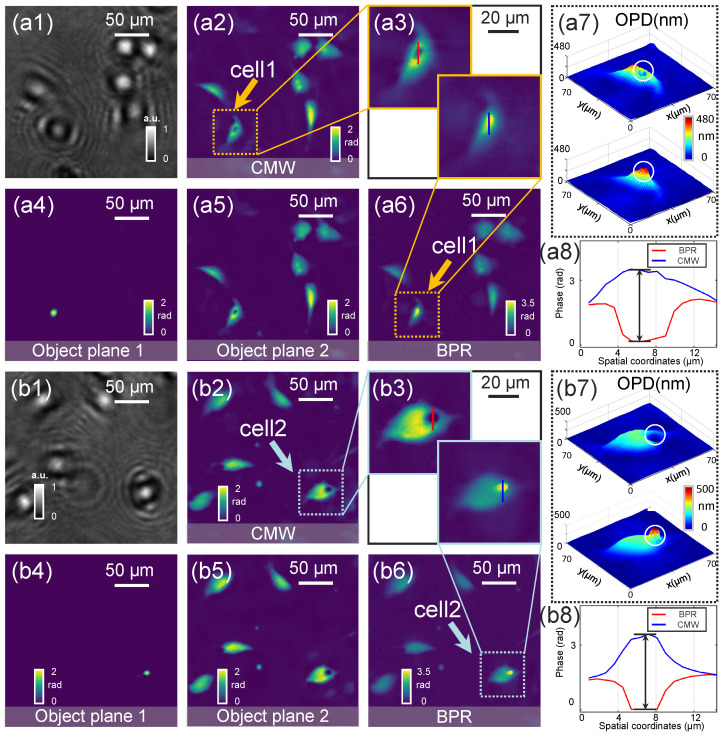
Experimental results of C166 cell slide. (**a1**,**b1**) The holograms of two selected regions. (**a2**,**b2**) Phase reconstruction of two regions under the CMW algorithm. (**a3**,**b3**) Zoomed-in comparison of the reconstructed phases of cell1 and cell2 using the CMW algorithm and the BPR algorithm. (**a4**,**a5**) and (**b4**,**b5**) Phase recovery of object plane 1 and object plane 2 in the selected region by the BPR algorithm. (**a6**,**b6**) Phase reconstruction of two regions under BPR algorithm. (**a7**,**b7**) Comparison of 3D rendering of cell1 and cell2 under CMW algorithm and BPR algorithm. (**a8**,**b8**) Phase values along the vertical line in (**a3**,**b3**).

Morphological analysis of biological cells is critical for early cancer diagnosis, enabling the quantification of changes associated with malignant transformation, including alterations in morphology, membrane dynamics, and refractive index. To compare the performance of the conventional multi-wavelength (CMW) phase recovery method [37] and BPR method for the detection of cell morphology, experiments were performed on C166 slides. Figure 3 depicts the comparative reconstruction results of C166 slides using both methods. Two selected regions are illustrated in Figure 3(a1,b1). In Figure 3(a2,b2), we illustrate the reconstructed phase using the CMW phase recovery method. Figure 3(a4–a6,b4–b6) show the phase recovery results of the BPR method for two selected regions of the cells. Two representative subregions (yellow and blue boxes) contain one individual cell, magnified in Figure 3(a3,b3). The proposed BPR method effectively resolves large phase values in cell imaging, producing high-accuracy phase reconstructions without artifacts such as the black spots observed in conventional methods. The phase values along the red and blue straight lines were extracted to plot quantitative phase curves, as shown in Figure 3(a8,b8). In both figures, the blue phase curve accurately reflects the true cell morphology. In contrast, the red phase curve exhibits a significant degradation, particularly in the concave region, where the phase of the large phase cell is not correctly recovered. Figure 3(a7,b7) illustrate a comparison of the three-dimensional (3D) morphology (refractive index accumulation over the cell thickness) of the cells in the two subregions. It can be found that the BPR method demonstrates a precise 3D morphology structure and accurately recovers the quantitative features of the sample.

### 3.2. BPR-LFOCM on QPI Validation

**Figure 4:**
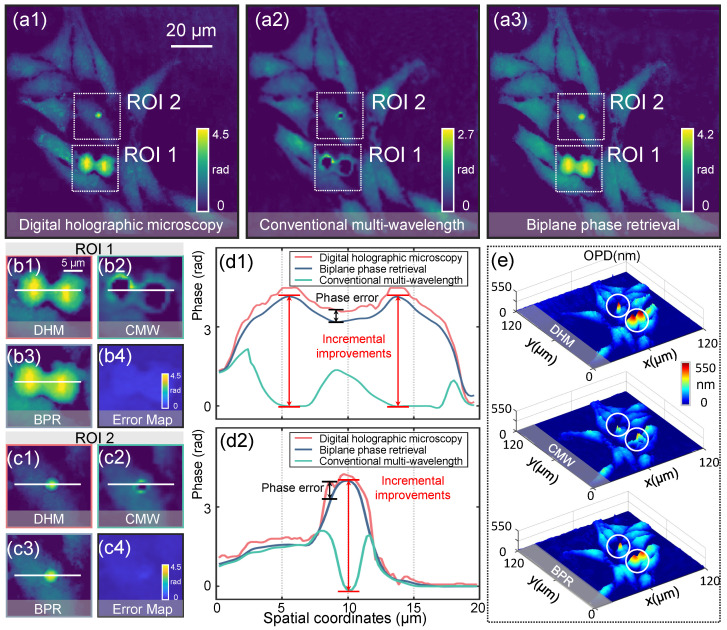
Experimental results of HeLa cells. (**a1**–**a3**) Phase reconstructed using DHM, CMW, and BPR methods. (**b1**–**b3**) Magnified regions of ROI 1. (**c1**–**c3**) Magnified regions of ROI 2. (**b4**,**c4**) Error maps between the BPR method and the DHM method in ROI 1 and ROI 2. (**d1**,**d2**) Phase value curves along the white solid line in ROI 1 and ROI 2. (**e**) A 3D rendering of the reconstructed phases by the DHM, CMW, and BPR methods.

The accuracy of the BPR method was validated using off-axis digital holographic microscopy (DHM) [42], which serves as the gold standard for QPI.In the DHM experimental system, the wavelength of illumination used is 532 nm. The light beam is transmitted through the objective lens (UPLanSAPO × 20/0.5NA, Olympus, Tokyo, Japan) and recorded by a camera (Imaging Source DMK 23 U274, 1600 × 1200, 4.4 µm). The reconstruction results of HeLa cells obtained by DHM are shown in Figure 4(a1). Comparison experiments were conducted in the same area using the LFOCM system, with all experimental conditions set according to the system parameters described in Figure 1. The cell phase is reconstructed and up-sampled for pixel matching with the DHM images using the CMW phase recovery method (Figure 4(a2)) and the BPR method (Figure 4(a3)). Figure 4(b1–b3) present captured images of HeLa cells undergoing cytoplasmic division, corresponding to the magnified ROI 1 region in Figure 4(a1–a3). Similarly, Figure 4(c1–c3) depicts the presence of large phase protrusions within the cells, corresponding to the amplified ROI 2 region in Figure 4(a1–a3). The phase values along the white line are extracted and plotted as quantitative curves in Figure 4(d1,d2), showing that the phase results recovered by the BPR method (blue curve) closely match those recovered by DHM, the gold standard (red curve). Comparison experiments using HeLa cells demonstrate that the BPR method achieves a phase accuracy of approximately 93%, significantly outperforming CMW phase recovery techniques. The 3D reconstruction outcomes under the three methods are presented in Figure 4e, further illustrating the accuracy of our method.

### 3.3. BPR-LFOCM on Live-Cell Culture

**Figure 5:**
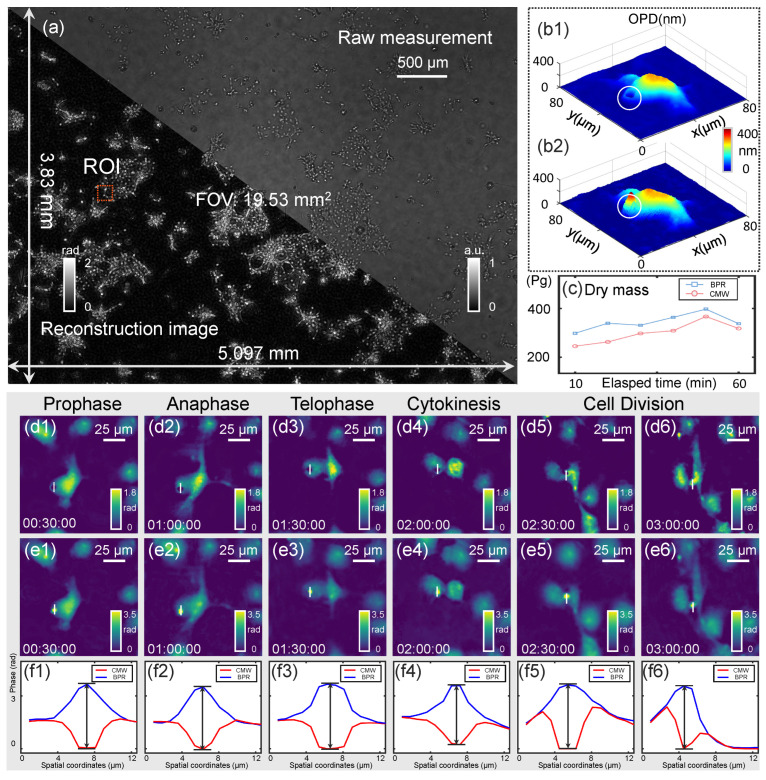
Dynamic phase imaging of COS-7 cells. (**a**) The hologram and phase reconstruction of the full FOV. (**b1**,**b2**) The 3D renderings corresponding to the cell in (**d1**,**d2**). (**c**) Cell dry mass computed under the BPR algorithm versus the CMW algorithm over time. (**d1**–**d6**,**e1**–**e6**) Six selected time-lapse phase images of ROI under the CMW algorithm and BPR algorithm. (**f1**–**f6**) Phase values along the white lines in (**d1**–**e6**).

Live-cell imaging experiments were conducted on COS-7 cells to evaluate the long-term stability of the BPR method under dynamic growth conditions. Our method can more accurately quantify the morphology of live cells and enhance imaging quality. As shown in Figure 5, the effectiveness of the BPR method was validated through *in vitro* experiments on unstained COS-7 cells conducted over a three-hour period.

The CMW phase recovery method (Figure 5(d1–d6)) and the proposed BPR method (Figure 5(e1–e6)) were employed to reconstruct the phase of the COS-7 cells in long-term live-cell culture, respectively. Our compact system allowed for *in situ* observation by placing it directly in the incubator, enabling quantitative analysis of the morphology of living cells throughout the FOV. The results show the process of cell mitosis [corresponding to the ROI 1 indicated by the red square]. The initial stages of chromatin aggregation in the pre-division phase of the cell are illustrated in Figure 5(d1,e1). Subsequently, we further selected one cell to study its morphology, and the pseudo-3D morphology of this cell was demonstrated under the two algorithms in Figure 5(b1,b2), respectively. It can be found that the BPR method demonstrates a more significant cell thickness, which provides more accurate morphological detection data for cell analysis. Figure 5(d2,e2) illustrate the mid-stage chromosomes divided into two groups and moved to opposite sides of the cell, and the cell was elongated. As illustrated in Figure 5(d3,e3), the cytoplasm split and the cell wall contracted and fell off. The subsequent stages, illustrated in Figure 5(d4,e4), demonstrate the emergence of individualized daughter cells. Moreover, the phase obtained from both methods is utilized to quantify the changes in dry mass throughout the cell division process (Figure 5c). The blue curve represents the results from the BPR method, which accurately tracks the changes in dry mass. In contrast, the red curve, resulting from the CMW method, exhibits discrepancies due to inaccuracies in phase recovery, leading to a partial loss of mass information. In Figure 5(f1–f6), phase values are extracted along the white lines shown in the corresponding cell images and plotted as quantitative curves. The figures clearly demonstrate the BPR method accurately reconstructed large phase changes during cell division, providing detailed morphological and quantitative phase data that conventional methods failed to resolve. This emphasizes the accuracy and reliability of the BPR method in QPI. Through the experimental results demonstrated in Figure 5, we can see the proposed BPR method could realize continuous analysis of large phase cells in the full FOV for long-term imaging, which provides a stable solver to achieve accurate reconstruction of large phase samples for live-cell research in the biomedical field.

## 4. Conclusions

In conclusion, this study introduced a BPR method for LFOCM, enabling precise QPI of large phase objects. We demonstrated the accuracy of the proposed method for QPI by comparing the results of large phase HeLa cell samples captured by off-axis DHM. Experiments on C166 cell slides and COS-7 cell cultures have shown that the BPR method can be applied to high-precision phase characterization and measurement of large phase samples, accurately reflecting the morphological data of cells. This will provide strong support for subsequent cell morphology and cell mass analysis. Compared with the conventional LFOCM phase recovery approach, the proposed method can more accurately recover the phase consistent with the nominal value. The BPR method demonstrates significant potential for high-throughput biomedical applications, particularly in live-cell imaging and analysis. A possible direction for future work is to utilize automatic focusing algorithms to adjust the reconstruction distance, thereby achieving complete automation of the imaging process and enhancing the overall efficiency of imaging.

## Data Availability

The data underlying the results presented in this paper are not publicly available at this time but may be obtained from the authors upon reasonable request.
